# A CRITICAL EXAMINATION OF AAA SERVICES AND SUPPORTS FOR CAREGIVERS IN MICHIGAN: POLICY AND PRACTICE IMPLICATIONS

**DOI:** 10.1093/geroni/igad104.1515

**Published:** 2023-12-21

**Authors:** Joan Ilardo, Angela Zell

**Affiliations:** Michigan State University, East Lansing, Michigan, United States; Michigan State University, East Lansing, Michigan, United States

## Abstract

To identify the service gaps for family caregivers in Michigan, this study examined the perceptions of stakeholders from the 16 area agencies on aging (AAA). An online questionnaire was administered to assess a point-in-time picture of current caregiver assessments and services in 2020. Fifteen AAAs responded to see definitely or probably an increase in the number of caregivers in their catchment areas. Fifteen AAAs currently purchase or contract for respite care services. Fourteen provide some type of direct caregiving resources. Twelve utilize staff to develop and monitor care plans. While some provided caregiver training, none directly provided supportive services. AAAs anticipated rapidly increasing needs for caregiver supports and services. While each provided a different array of services, they anticipated that demand would continue to increase given a rising aging population in Michigan. Results were used to craft a proposal to establish a network of regional caregiver resource centers. Recommendations in the study include creating a task force of stakeholders to identify standardized service definitions, measures and reporting requirements; create a no-wrong-door, multi-prong approach for caregivers to access programs; provide respite care through contracts and referrals to community-based organizations; create a network of trainers to disseminate evidence-based best practices to AAAs and contractors; use untapped resources such as AARP and Alzheimer’s Association; explore forming local healthcare systems and provider collaborations to identify caregivers and triage them. Next steps are to pursue funding from the legislature and other sources to create regional caregiver resource centers across the state.

